# High resolution mapping of trypanosomosis resistance loci *Tir*2 and *Tir*3 using F12 advanced intercross lines with major locus *Tir*1 fixed for the susceptible allele

**DOI:** 10.1186/1471-2164-11-394

**Published:** 2010-06-22

**Authors:** Joseph K Nganga, Morris Soller, Fuad A Iraqi

**Affiliations:** 1 International Livestock Research Institute, P. O. Box 30709, Nairobi, Kenya; 2 Jomo Kenyatta University of Agriculture and Technology, P.O. Box 62000, Nairobi, Kenya; 3 Department of Genetics, Hebrew University, Jerusalem, Israel; 4 Department of Clinical Microbiology and Immunology, Sackler Faculty of Medical, Tel Aviv University, Tel Aviv, Israel

## Abstract

**Background:**

Trypanosomosis is the most economically important disease constraint to livestock productivity in Africa. A number of trypanotolerant cattle breeds are found in West Africa, and identification of the genes conferring trypanotolerance could lead to effective means of genetic selection for trypanotolerance. In this context, high resolution mapping in mouse models are a promising approach to identifying the genes associated with trypanotolerance. In previous studies, using F2 C57BL/6J × A/J and C57BL/6J × BALB/cJ mouse resource populations, trypanotolerance QTL were mapped within a large genomic intervals of 20-40 cM to chromosomes MMU17, 5 and 1, and denoted *Tir*1, *Tir*2 and *Tir*3 respectively. Subsequently, using F6 C57BL/6J × A/J and C57BL/6J × BALB/cJ F6 advanced intercross lines (AIL), *Tir*1 was fine mapped to a confidence interval (CI) of less than 1 cM, while *Tir*2 and *Tir*3, were mapped within 5-12 cM. *Tir*1 represents the major trypanotolerance QTL.

**Results:**

In order to improve map resolutions of *Tir*2 and *Tir*3, an F12 C57BL/6J × A/J AIL population fixed for the susceptible alleles at *Tir*1 QTL was generated. An F12 C57BL/6J × A/J AIL population, fixed for the resistant alleles at *Tir*1 QTL was also generated to provide an additional estimate of the gene effect of *Tir*1. The AIL populations homozygous for the resistant and susceptible *Tir*1 alleles and the parental controls were challenged with *T. congolense *and followed for survival times over 180 days. Mice from the two survival extremes of the F12 AIL population fixed for the susceptible alleles at *Tir*1 were genotyped with a dense panel of microsatellite markers spanning the *Tir*2 and *Tir*3 genomic regions and QTL mapping was performed. *Tir*2 was fine mapped to less than 1 cM CI while *Tir*3 was mapped to three intervals named *Tir*3a, *Tir*3b and *Tir*3c with 95% confidence intervals (CI) of 6, 7.2 and 2.2 cM, respectively.

**Conclusions:**

The mapped QTL regions encompass genes that are vital to innate immune response and can be potential candidate genes for the underlying QTL.

## Background

Trypanosome infection (Trypanosomosis) is a disease of man, domestic livestock and wildlife. Trypanosomosis is the most economically important disease constraint to livestock productivity in Africa [1,2]. The major pathogenic trypanosomes transmitted by the tsetse fly include *T. congolense*, *T. vivax, T. brucei*, and *T. evansi*, which cause the disease either as single or multiple infections. The disease is characterized by anemia as evidenced by reduction in packed cell volume due to erythrophagocytosis) fever, weight loss, fatigue and heart failure [3]. This results in loss of production, and in more severe cases, death of the animal [4]. Certain breeds of cattle show a remarkable resistance to the effects of trypanosomosis. This phenomenon is termed 'trypanotolerance' because the host tolerates the presence of the parasites, while not showing the severe anemia and production loss that characterize the infection in susceptible breeds [5]. Selective breeding of livestock for trypanotolerant traits would provide a partial solution to livestock based agriculture in tsetse-infested areas [3]. Recently, quantitative trait loci (QTL) associated with trypanotolerance traits in cattle were reported [6].

Parallel to QTL mapping in cattle, experimental studies in mice have also been used to map trypanotolerance QTL [7,8]. The mouse offers a powerful model for studying the genetics of disease resistance [9]. Inbred strains of mice show different responses to trypanosomosis [10], and different capacity of controlling anemia during infection [11]. In particular, the C57BL/6J strain of inbred mice presents a higher degree of resistance to *T. congolense *than the A/J and BALB/cJ mouse strains [10,12]. Trypanotolerance in mice is related to early control of parasitemia [13], a capacity that is associated with genes that are expressed early in the course of the infection. These early genes regulate parasite growth and determine how rapidly the immune response is triggered. Susceptible mouse strains show sustained high levels of parasitemia after challenge by *T. congolense *whereas low levels are shown by the resistant strains [14]. Thus, mapping QTL affecting trypanotolerance in mice provides an alternative approach to identifying the genes associated with trypanotolerance.

In previous studies of survival time following *T. congolense *challenge in F2 crosses between the susceptible A/J and BALB/cJ strains and the resistant C57BL/6J mice, three trypanosomosis resistance QTL (denoted, *Tir*1, *Tir*2 and *Tir*3) were identified and mapped to large genomic intervals on mouse chromosome MMU17, MMU5 and MMU1 respectively [8]. *Tir*1 represents the major trypanotolerance QTL with an additive effect of 31 days on survival time [8]. Following the initial QTL mapping results, the advanced intercross lines (AIL) approach [15] was exploited for further fine mapping of trypanotolerance QTL in mice [16]. Using F6 C57BL/6J × A/J and C57BL/6J × BALB/cJ AIL populations, *Tir*1 was indeed mapped to a 95% confidence interval (CI) of 1.3 cM (17.3--18.6 cM) on MMU17 [16]. In the same AIL, however, *Tir*2, and *Tir*3 were only mapped to much larger CIs: *Tir*2, to a 95% CI of 12 cM (39-51 cM), on MMU5; while *Tir*3 resolved into three distinct peaks on MMU1, each representing a different putative QTL (denoted, *Tir*3a, *Tir*3b and *Tir*3c). These three QTL mapped to 95% CIs of 10 cM (68-78 cM), 1.8 cM (58.8-60.6 cM) and 8 cM (90-98 cM) on MMU1, respectively [16]. Thus, high resolution mapping of *Tir*2 and *Tir*3 to a small genomic confidence interval, as required for positional cloning of genes underlying the QTL remained to be achieved.

Here, we report further fine mapping of *Tir*2 and *Tir*3, using an F12 AIL fixed for the susceptible allele at *Tir*1. The underlying rationale is that fixing *Tir*1 would reduce residual ("error") variance and hence result in stronger relative effects of *Tir*2 and *Tir*3, while the use of an F12 AIL would increase recombination events facilitating fine mapping. To fully exploit the additional recombination events at the F12 AIL, it was necessary to employ a denser set of markers than those used in F6 mapping. Hence, selective genotyping [17] was employed in order to reduce the associated genotyping load. This procedure has been shown to reduce genotyping load with little reduction in the power of QTL detection [17].

## Results

### Line comparisons

**Figure 1:**
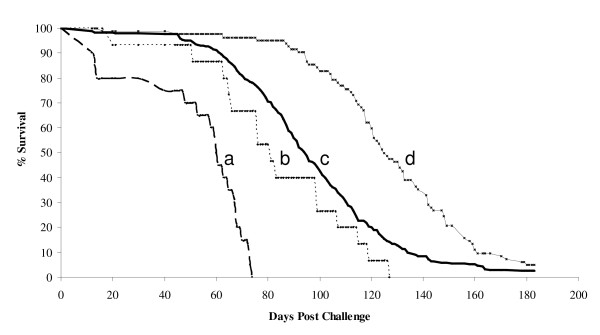
**Percentage survival over time following *T. congolense *challenge of (a) A/J, (b) C57BL/6J, (c) F12 AIL fixed for A/J alleles on *Tir1 *QTL (D17SS) and (d) F12 AIL fixed for C57BL/6J alleles on *Tir1 *(D17RR)**.

The C57BL/6J (resistant) and the A/J (susceptible) parental populations had mean survival times of 81 and 60 days, respectively; while mean survival times of D17RR (fixed for resistant allele at *Tir*1) and D17SS F12-AIL (fixed for susceptible allele at *Tir*1) were 125 and 95 days respectively (Figure 1). The range in survival time was about 100 days for C57BL/6J, and the two F12 lines, but somewhat less (80 days) for the A/J susceptible line. All differences between lines in mean survival time were statistically significant (P < 0.01). Both the comparison of parental C57BL/6J to A/J and of D17RR to D17SS confirm that the challenge protocol was effective in distinguishing between resistant and susceptible animals. Note that the D17SS F12-AIL is also the mapping population used in the present study. The difference of 30 days in mean survival time between D17SS and D17RR accords well with the estimated effect of this locus in the F2 and F6 experiments reported previously (Table 1). The mean survival time of the D17SS line, which is homozygous for the susceptible allele at the strong *Tir*1 QTL on MMU17, was somewhat greater than that of the resistant C57BL/6J mice. This will be addressed in the Discussion section.

**Table 1:** Summary table of QTL comparing results of the F2^1^, F6^2 ^and F12^3 ^AIL mapping populations.

*Tir*	MMU		**F2**^**1,7**^	**F6 AIL**^**2,**^	**F12 AIL**^**3**^
*Tir*1	17	Map Location (cM)	~14	17.9 cM	ND

		CI (cM)	ND	0.9 cM	ND

		Effect (days)	33.5	38.8	30.0

		PVar (%)	11.8	19.6	ND

Tir2	5	Map Location (cM)	~20	45	41

		CI (cM)	ND	12	1

		Effect (days)	22	14.7	8.9

		PVar (%)	5.3	3.3	4.5

*Tir*3a	1	Map Location (cM)		59.7	67

		CI (cM)		1.8	6

		Effect (days)		14.45	11.33

		PVar (%)		2.6	6.7

*Tir*3b	1	Map Location (cM)	73	73	76

		CI (cM)	ND	10	7

		Effect (days)	32	17.3	11.3

		PVar (%)	8.9	4.1	7.9

*Tir*3c	1	Map Location (cM)		94	86

		CI (cM)		8	2

		Effect (days)		13.4	12.1

		PVar (%)		2.3	8.6

The most likely map locations (cM) are given as distances in cM from the telomere. Effect, difference between alternative homozygous genotypes (additive effect) in days^4^; CI, 95% confidence intervals for QTL map location in cM^5^; PVar, proportion of phenotypic variance explained by each of the *Tir *loci^6^.

^1 ^Data from [7]; Mean of BALB/cJ × C57BL/6J and A/J × C57BL/6J.

^2 ^Data from [16,19].

^3 ^Generated from A/J × C57BL/6J and fixed for MMU17 A/J (susceptible) allele.

^4 ^For F12 AIL, dominance effects are *Tir*2, 4.5; *Tir*3a, -1.2; *Tir*3b, -5.7; *Tir*3c, -5.0.

^5 ^For F12 AIL, flanking markers defining the most likely interval containing the QTL are: *Tir*2, D5MIT258-58; *Tir*3a, D1MIT94-140; *Tir*3b, D1MIT288-105; *Tir*3c, D1MIT107-58.

^6 ^For F12 AIL, LOD scores are: *Tir*2, 3.81; *Tir*3a, 4.49; *Tir*3b, 3.48; *Tir*3c, 3.18.

^7 ^*Tir*3 mapped as a single locus in the F2 experiment.

### Marker positions and map expansion

Table 2 (MMU1) and Table 3 (MMU5) show marker position and marker-marker distances based on the F12 marker genotypes. Marker order in the F12 map was the same as for the MGD map, but map distances, as expected, were much greater. Map distance for the region examined on MMU1 extended from D1MIT49 (at 54.5 cM on the MGD map) to D1MIT17 (106.3 cM on the MGD map), a total of 51.8 cM. This region expanded 11.8-fold to a total of 607.7 cM on the F12 map. Similarly, map distance for the region examined on MMU5 extended from D5MIT184 (at 33 cM on the MGD map) to D1MIT169 (at 86 cM on the MGD map), a total of 53 cM. This region expanded 9.1-fold to a total of 485.4 cM on the F12 map. Both of these map expansions were greater than the 6-fold expansion expected for an F12 AIL [15]. All genotypes were carefully double-checked. Map expansion is expected to vary among chromosomal regions due to chance sampling of recombination break-points accumulated in each region across the generations of AIL development. This is particularly true when the AIL is developed on the basis of a rather small number of individuals each generation. This was the case for the F12, particularly for the number of individuals sampled in the F9 to F12 generations. Since only two regions were examined, sampling can also be the reason that both regions showed the same direction of deviation from expected. Correlations between marker-to-marker map intervals in the MGD map and the F12 AIL map were 0.03 (NS) for MMU1 and 0.26 (P = 0.10) for MMU5.

**Table 2:** MMU1, marker position (cM and Mbp) and marker to marker distances on the MGD and F12 linkage maps.

MGD linkage map			F12 linkage map	
**Marker order**	**Position (cM)**	**Distance in cM**	**Position (cM)**	**Distance in cM**

D1MIT49	54.5		0	
D1MIT60	58.7	4.2	40.3	40.3
D1MIT87	62.1	3.4	93.8	53.5
D1MIT217	63.1	1	125.5	31.7
D1MIT94	64.0	0.9	147.2	21.7
D1MIT139	65.0	1	158.3	11.1
D1MIT286	67.0	2	179.6	21.3
D1MIT140	70.0	3	203.9	24.3
D1MIT288	71.5	1.5	272.9	69.0
D1MIT102	73.0	1.5	302.7	29.8
D1MIT105	80.0	7	324.3	21.6
D1MIT425	81.6	1.6	337.3	13.0
D1MIT107	85.0	3.4	351.7	14.4
D1MIT16	87.2	2.2	413.8	62.1
D1MIT36	92.3	5.1	435.1	21.3
D1MIT356	95.8	3.5	494.8	59.7
D1MIT355	97.0	1.2	529.2	34.4
D1MIT403	100.0	3	545.4	16.2
D1MIT165	100.0	0	561.1	15.7
D1MIT221	102.0	2	582.6	21.5
D1MIT17	106.3	4.3	607.7	25.1

**Total Distance**		**51.8 cM**		**607.7 cM**

**Table 3:** MMU5, marker position (cM and Mbp) and marker to marker distances on the MGD and F12 linkage maps.

MGD linkage map			F12 linkage map	
**MGD Marker order**	**Position (cM)**	**Distance in cM**	**Position (cM)**	**Distance in cM**

D5MIT184	33.0		0	
D5MIT255	34.0	1	22.5	22.5
D5MIT200	36.0	2	73	50.5
D5MIT258	41.0	5	107.4	34.4
D5MIT58	41.0	0	129.2	21.8
D5MIT201	42.0	1	141	11.8
D5MIT20	52.0	10	170.4	29.4
D5MIT172	53.0	1	193.4	23.0
D5MIT10	54.0	1	209.9	16.5
D5MIT157	57.0	3	227.7	17.8
D5MIT240	59.0	2	265.2	37.5
D5MIT24	60.0	1	297.4	32.2
D5MIT188	64.0	4	317.8	20.4
D5MIT242	66.0	2	329.1	11.3
D5MIT95	68.0	2	338.1	9.0
D5MIT372	73.0	5	374.3	36.2
D5MIT168	78.0	5	405.7	31.4
D5MIT375	81.0	3	425.7	20.0
D5MIT223	84.0	3	453.7	28.0
D5MIT169	86.0	2	485.4	31.7

**Total Distance**		**53 cM**		**485.4**

### LOD score plots

**Figure 2:**
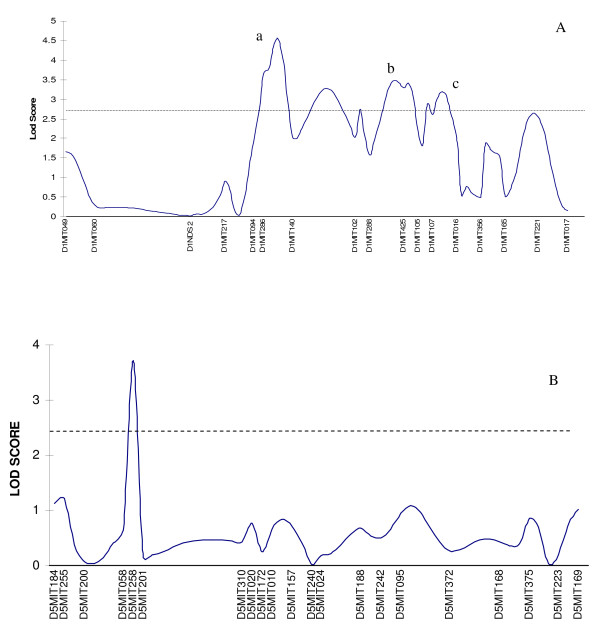
**(A) Likelihood plot across *Tir*3 region and (B) Likelihood plot across *Tir*2 region**. (A) LOD scores associated with microsatellite markers on MMU1 are shown for the genotyped F12 D17SS AIL. Only marker driven peaks above the experimental threshold labeled as a, b and c were accepted as putative QTL with the others being disregarded. (B) LOD scores associated with microsatellite markers of MMU5 are shown for the genotyped F12 D17SS AILs.

Threshold LOD scores for significance based on permutation tests [18] were 2.45 for MMU1 and 2.67 for MMU5. Figure 2 shows LOD score plots for MMU1 and MMU5 obtained by the MapMaker QTL software. MMU5 presented a single very sharp LOD score peak exceeding the significance threshold. This peak closely corresponds to the QTL *Tir*2 identified in previous studies. MMU1 presented four LOD score peaks that exceeded the significance threshold. However, only the three peaks designated a, b, and c in the figure, were declared significant by MapMaker QTL. These peaks are taken to correspond to the QTL *Tir*3a, *Tir*3b, and *Tir*3c, identified in previous studies. The fourth peak (located between the peaks labeled a and b in the figure) was not declared significant by MapMaker QTL (nor by QTL Express, data not shown). We attribute this to the absence of any significant single-marker test across the region of this peak, as defined by flanking markers D1MIT204 and D1MIT102.

### Effects of the *Tir *QTL

Table 1 shows position and effects of *Tir*2 and the three *Tir*3 QTL as were estimated from the F12-AIL. The three *Tir*3 loci had roughly equal effect, with the susceptibility allele at each of the loci reducing survival time by 11 to 12 days. *Tir*3a was almost completely additive, while *Tir*3b and *Tir*3c showed partial dominance in the direction of resistance (dominance ratios 0.51 and 0.42, respectively). Total summed additive effect of all three *Tir*3 loci was 34.64 days. The effect of *Tir*2 (8.91 days; 4.5% of variance explained)) was quite a bit less than that of the *Tir*3 loci; and in contrast to *Tir*3, dominance at *Tir*2 was in the direction of susceptibility.

### Locations and confidence intervals of the *Tir *QTL

*Tir*2 mapped to a confidence interval of 1 cM in the present study, at 41 cM on MMU5. This is comfortably within the region 39 - 51 cM with peak at 44 cM found in the previous F6 AIL study [16] so that the two experiments are almost certainly identifying the same QTL. The CI of 1 cM obtained in the present study is a distinct improvement on the CI of 12 cM obtained in the F6 study.

The F12 mapping results fully confirmed the complex tripartite substructure of *Tir*3 as hinted at in the F2 mapping results and clearly expressed in the F6 AIL [16]. However, correspondence of the F6 and F12 mapping results was not as close for the three *Tir*3 loci as for *Tir*2, and the reduction in CI was for the most part marginal. For *Tir*3a, the F12 CI (64 - 70 cM) was distal to the corresponding F6 CI (58.8 - 60.6 cM) and considerably larger (6 cM and 1.8 cM, respectively). For *Tir*3b, the F12 CI (72.2 -- 79.3 cM) corresponded closely to the *Tir*3b F6 CI (68 - 78 cM). However, improvement in CI was slight (from 10 cM in the F6 to 7 cM in the F12). For *Tir*3c, F12 CI (85-87.2 cM) was proximal to the F6 CI (90-98 cM), but was considerably narrower (2 cM and 8 cM, respectively). Thus, *Tir*3b which is the central QTL in this region mapped to the same location as in the F6, while *Tir*3a and *Tir*3*c *both appear to have moved toward the center. We return to this in the Discussion.

## Discussion

Parental C57BL/6J mice had a 21 day higher mean survival time than parental A/J mice. This agrees with the purported higher resistance of the C57BL/6J compared to A/J mice. However, the absolute magnitude of the difference was much less than the 75 days previously reported [8]. We believe that this is best explained by factors associated with the source supplier of the parental lines. Variation in the *T. congolense *clones might also be considered, but this would be expected to affect the estimated effects at the *Tir*1 locus as well. This was not found; allele substitution effects at *Tir*1 were similar in all three experiments.

In addition, in the original study of the F2 of a cross between these two lines [8] the F2 was intermediate in resistance between the two parental lines; while in the present study the D17SS AIL (fixed for the susceptible allele at *Tir*1) was more resistant than the resistant C56BL/6J parent line. The F6 AIL also displayed higher resistance than the C56BL/6J parent line (data not shown). A genetic explanation for these results is not evident, and hence we believe they too should be attributed to some factor associated with the source supplier of the parental lines. A further possibility is an accumulated effect of environmental factors on the AIL populations that improved their phenotypic response to the infection, especially considering that the AIL population were developed during a period of three years at ILRI in an open environment mouse facility. More speculatively, this increase might also be due to dissociation of suppressor factors linked to host resistant genes due to increase in the recombination events in AIL generations. Such a dissociation might lead to an increase in the expression of resistant phenotypes in these mouse populations.

Table 1 summarizes the results of the present study, and of the previous F2 [8] and F6 AIL [16,19] studies of these lines. With minor exceptions, the results are quite consistent across the three studies. *Tir1 *mapped to same general location in F2 and F6 AIL (17.9 cM), with very narrow CI, and hence was not mapped again in the F12 AIL. Effect of the locus on 50% survival time was consistent in all three studies (33.5, 38.8 and 30.0 days for the F2, F6 AIL and F12 AIL, respectively. *Tir*2 mapped to the same general location in F6 and F12 AIL but more proximally in the F2. Estimate of effects differed across the three studies, from 22, 14.7 and 8.9 days, respectively. Estimate of location had very narrow CI in the F12 AIL. *Tir*3 mapped to the same general location in all three studies, but was resolved into three distinct peaks in the two AIL analyses. Summed effect of the *Tir*3 chromosomal region was similar in all three studies: 32.0, 45.2 and 34.64, for F2, F6 AIL, and F12 AIL, respectively. The proportion of phenotypic variance explained by *Tir*2, and *Tir*3c in the present study was comparable to that found in the F2 [8] and in the F6 AIL studies [16]. The proportion of variance explained by *Tir*3a and *Tir*3b in the present study, however, was considerably greater than found in the F6 AIL. Overall, the proportion of variance explained by *Tir*2 and *Tir*3 was somewhat greater in the present study than in the F6 AIL. This may be attributed to the fixing of the strong *Tir*1 locus, which contributed 11.8% and 19.6% of phenotypic variance in the F2 and F6 AIL populations, respectively. *Tir*3b mapped to about the same location in both the F6 and F12 AIL, but with fairly wide CI in both (10 cM and 7 cM, respectively). *Tir*3a and *Tir*3b in the F6 AIL, however, mapped a bit proximal (*Tir*3a) and a bit distal (*Tir*3c) to their F6 AIL locations, bringing them each somewhat closer to *Tir*3b in the F12. A narrow CI was achieved for *Tir*3a in the F6 AIL, and for *Tir*3c in the F12 AIL. Analysis of map order in the F6 AIL marker map showed a large number of discrepancies between the map order inferred from the F6 AIL genotypes, and the standard MGD map order of that time (Data not shown). In contrast, map order of the F6 and F12 AIL marker maps was fully consistent with the current MGD map. For this reason we favor the map locations of the QTL obtained in the F12 population.

Thus, of the four *Tir *QTL mapped in the F12 AIL, higher map resolution was achieved for *Tir*2 and *Tir*3*c*, but not for *Tir*3a or *Tir*3b. However, *Tir*3a had already been mapped to a narrow CI in the F6 AIL. Taken as a whole then, the AIL approach successfully resolved the complex *Tir*3 locus into three component loci, and together with *Tir*1 and *Tir*2 mapped four of the resultant five *Tir *loci (all except for *Tir*3b) to CI that are sufficiently narrow (<2 cM), to make positional cloning feasible. It is quite possible that combining the F6 and F12 data might yield higher resolution for *Tir*3b as well.

As an alternative to the AIL approach for high resolution mapping, it may have been possible to generate Interval Specific Congenic Lines [20] for each of the *Tir *QTL. These have been used successfully for high resolution of individual QTL, (see, e.g., [21,22]). However, in these cases, a single QTL was dissected. The present study involved five QTL; thus, five series of ICSL lines would have had to be developed. Logistically, the AIL seems to have delivered about the same degree of map resolution with far less resources.

A number of candidate trypanotolerance genes within the fine mapped *Tir*2 loci were identified from the public databases http://www.informatics.jax.org/. We have identified forty three genes mapped within the fine mapped intervals of *Tir*2 QTL (between D5MIT258-D5MIT58). Of these, the most attractive candidate genes which may underly the QTL are Toll-like receptor 1, Toll-like receptor 6, both of which are well known to be associated innate immunity and host response to infection diseases and are important for the elimination of invading pathogens. Toll-like receptors (*TLRs*) are members of the pattern-recognition receptor (*PRR*) family and play a central role in initiation of innate cellular immune response and subsequently adaptive immune response to pathogens. *TLRs *can recognize diverse pathogen-associated molecular patters (*PAMPs*) make it an early warning system against invading pathogens. Activation of *TRL *signal initiates pathways, which leads to activation of variety of genes that function in host defense mechanism, including those of inflammatory cytokines, chemokines, and antigen presenting molecules. These genes are the most relevance for our studied phenotype and can be involved in clearance of trypanosma parasite from the infected mice.

For the *Tir*3 region, attractive candidate genes include Interleukin 10 and its regulator *CYPr2 *gene on *Tir*3a and b respectively, and various tumor necrosis factor ligand super-family members, interferon related segments, and Toll like receptor 5 genes. These genes are involved in host response to various pathogenic infections hence may play a role in trypanotolerance.

## Conclusions

The cumulative results of the mouse F2 and AIL mapping studies provide essential mapping information for the identification of candidate trypanotolerance genes in mice, and may be useful for the identification of the homologous genes in livestock. Further analysis of these and other candidate genes is vital in order to ascertain their role in trypanotolerance. In future studies, we plan to examine the candidate genes for suggestive polymorphisms, and perform expression assays for these and other candidate genes under control and challenge conditions as the next step to identify the actual genes involved in resistance.

## Methods

### Development and selection of *Tir1 *fixed lines

Experimental lines fixed for the susceptible allele at *Tir*1 were generated by genotyping 200 mice of each sex from the F9 C57BL/6J × A/J AIL with a panel of 12 microsatellite markers spanning the *Tir*1 QTL region from 15.9 cM (D17Mit29) to 21.95 cM (D17Mit11). Males and females of the F9 AIL population were selected depending on whether they were homozygous for the chromosomal haplotypes representing the susceptible or resistance alleles at *Tir*1, with the rest of the genome being random. These animals (10 breeding pairs of each designated genotype) were intermated by genotype to produce two lines, one (designated D17SS), putatively homozygous for the susceptibility allele at *Tir*1 and the other (designated D17RR), putatively homozygous for the resistance allele. In order to expand the population, the F9 *Tir*1 homozygous lines were then intermated for three further generations to produce 600 and 100 of D17SS and D17RR F12 mice respectively. D17SS and D17RR were generated at the International Livestock Research Institute (ILRI) small animal facility (SAU). In addition, 30 animals each of the two parental inbred mouse lines, A/J and C57BL/6J, were purchased from Harlan UK Ltd., (Bicester, Oxon, U.K). At age of 12 weeks, all animals (i.e., the 600 D17SS and 100 D17RR F12 animals, and the 30 mice of each of the parental inbred lines, A/J and C57BL/6J), were challenged by intraperitoneal injection of 10^4 ^bloodstream forms of *T. congolense *clone 1180 in a total volume of 200 μl phosphate-buffered saline glucose [16]. Survival time for each individual was recorded over a period of 180 days, with mice surviving longer than 180 days, being assigned a survival time of 180 days. Differences between mean survival time of the various lines were tested for significance by t-test, using R/4 as an estimate of the SD of survival time within each line, where R is the range that includes 95% of the individuals in each line, averaged over all four lines. All experimental mice and protocols were approved by the Institutional Animal Care and Use Committee of ILRI. During the experiment, mice were housed on hardwood chip bedding in cages at an open environment animal facility and were given tap water and rodent chow *ad libitum*. The parental and D17RR lines were included in the experiment to serve as a positive control for the challenge protocol. Studies in mice show that animals of the same inbred strain reared in different facilities, behave differently when tested by the same protocol (see, e.g., [23]. Consequently, since the F12 animals and the parental lines were produced under different conditions, and are separated by many generations from the time the AIL was initiated, it is not appropriate to make direct comparisons between the survival curves of the parental lines and the F12 populations.

### Microsatellite typing

One hundred mice representing 16.5% of the 600 *T. congolense*-inoculated F12 D17SS mice were selected from each of the phenotypic extremes for genotyping (total, 200 mice). These were the first 100 and the last 100 D17SS mice to succumb, including mice that survived the full 180 days. Fluorescent dye labeled microsatellite markers (Research Genetics, Huntsville Ala USA) within the previously mapped *Tir*2 and *Tir*3 QTL regions on MMU1 and MMU5 were selected for genotyping from the mouse genome databases at http://www.broadinstitute.org/science/projects/mammals-models/mouse/mouse-genome-links. At MMU1 an additional 13 markers were genotyped, giving a total of 21 markers covering 51.8 cM, with mean marker interval of 2.59 cM; while at MMU5 an additional 14 markers were genotyped, giving a total of 20 markers covering 53.0 cM, with mean marker interval of 2.79 cM (see Figure 2 for details). Tables 2 and 3 show all markers used, and their location on the MGD marker map. Marker order was the same on the EMSEMBL marker map, with the exception of D1MIT102 and D1MIT288 whose order is reversed on the EMSEMBL map, relative to the MGD map.

Mouse genomic DNA was extracted from tail tissue by standard phenol chloroform extraction [24] and suspended in Tris EDTA buffer (pH 7.6). PCR amplification was carried out in a DNA thermal cycler 9600 (Perkin-Elmer Cetus, Norwalk, Conn.) as described earlier [8]. Fluorescent PCR products were loaded and separated on automated DNA sequencers (ABI PRISM^® ^377, Applied Biosystems). The genotypes were assigned using Genescan^® ^672 (Version 2.0.2 Applied Biosystems) and Genotyper^® ^(Version 2.0 Applied Biosystems) software.

### Linkage analysis and QTL mapping

Based on the F12 genotyping results, microsatellite markers used for fine mapping *Tir*2 and *Tir*3 loci on MMU1 and MMU5 were mapped and best ordered on each chromosome, using the MapMaker/QTL program [25]. MapMaker/QTL was also used for mapping on these chromosomes of QTL having an effect on mean survival time following *T. congolense *challenge. MapMaker/QTL provides an estimate of additive and dominance effects at each of the QTL, and the proportion of the phenotypic variance explained by each of the putative QTL. Threshold effects for declaring significance and bootstrap estimates of confidence intervals of QTL map location were obtained using the QTL Express program http://qtl.cap.ed.ac.uk/[26].

When selective genotyping is employed, estimates of QTL effect are biased upward [17]. However, unbiased estimates are obtained when maximum likelihood methods are employed that make use of the entire phenotypic distribution, including the animals that were not genotyped [27]. This is the procedure used by Mapmaker/QTL, and hence the results provided by this program are unbiased by the applied selection.

## Abbreviations

AIL: Advanced Intercross Line; CI: Confidence interval; QTL: Quantitative trait locus; TLR: Toll like receptor

## Authors' contributions

All authors read and approved the text. JKN was responsible for genotyping the mice, performing QTL mapping and drafting the initial manuscript. MS was responsible for reviewing the data and the supporting statistical analyses and QTL mapping results, and participated actively in writing and revising the manuscript. FAI was responsible of designing, generating, and challenging the F12 population, recording of the phenotypic data, designing the genotyping effort, reviewing the QTL mapping results and was active in preparing the manuscript.

## Acknowledgements

We wish to thank John Wambugu, Moses Ogugo, Daniel Mwangi and Nemwal Nyamweya from ILRI for their excellent technical assistance. Special thanks also goes to, Prof Mabel Imbuga of Jomo Kenyatta University of Agriculture and Technology (JKUAT) for her support and advice. We also wish to acknowledge the contribution JKUAT, the International Livestock Research Institute training unit under Dr Rob Eley for the graduate fellowship. This work was directly funded by the Welcome Trust, United Kingdom, Japan and European Union through the CGIAR.
